# Delays in reporting and publishing trial results during pandemics: cross sectional analysis of 2009 H1N1, 2014 Ebola, and 2016 Zika clinical trials

**DOI:** 10.1186/s12874-021-01324-8

**Published:** 2021-06-08

**Authors:** Christopher W. Jones, Amanda C. Adams, Elizabeth Murphy, Rachel P. King, Benjamin Saracco, Karen R. Stesis, Susan Cavanaugh, Brian W. Roberts, Timothy F. Platts-Mills

**Affiliations:** 1 Department of Emergency Medicine, Cooper Medical School of Rowan University, One Cooper Plaza, Suite 152, Camden, NJ 08103 USA; 2 Medical Library, Cooper Medical School of Rowan University, Camden, NJ 08103 USA; 3 Quantworks, Inc, 202 S. Greensboro St, Carrboro, NC 27510 USA

**Keywords:** Trial registration, Clinicaltrials.gov, Pandemic, H1N1, Ebola, Zika, Publication bias, Non-publication

## Abstract

**Background:**

Pandemic events often trigger a surge of clinical trial activity aimed at rapidly evaluating therapeutic or preventative interventions. Ensuring rapid public access to the complete and unbiased trial record is particularly critical for pandemic research given the urgent associated public health needs. The World Health Organization (WHO) established standards requiring posting of results to a registry within 12 months of trial completion and publication in a peer reviewed journal within 24 months of completion, though compliance with these requirements among pandemic trials is unknown.

**Methods:**

This cross-sectional analysis characterizes availability of results in trial registries and publications among registered trials performed during the 2009 H1N1 influenza, 2014 Ebola, and 2016 Zika pandemics. We searched trial registries to identify clinical trials testing interventions related to these pandemics, and determined the time elapsed between trial completion and availability of results in the registry. We also performed a comprehensive search of MEDLINE via PubMed, Google Scholar, and EMBASE to identify corresponding peer reviewed publications. The primary outcome was the compliance with either of the WHO’s established standards for sharing clinical trial results. Secondary outcomes included compliance with both standards, and assessing the time elapsed between trial completion and public availability of results.

**Results:**

Three hundred thirty-three trials met eligibility criteria, including 261 H1N1 influenza trials, 60 Ebola trials, and 12 Zika trials. Of these, 139 (42%) either had results available in the trial registry within 12 months of study completion or had results available in a peer-reviewed publication within 24 months. Five trials (2%) met both standards. No results were available in either a registry or publication for 59 trials (18%). Among trials with registered results, a median of 42 months (IQR 16–76 months) elapsed between trial completion and results posting. For published trials, the median elapsed time between completion and publication was 21 months (IQR 9–34 months). Results were available within 24 months of study completion in either the trial registry or a peer reviewed publication for 166 trials (50%).

**Conclusions:**

Very few trials performed during prior pandemic events met established standards for the timely public dissemination of trial results.

**Supplementary Information:**

The online version contains supplementary material available at 10.1186/s12874-021-01324-8.

## Background

As the coronavirus 2019 (COVID-19) pandemic has escalated across the globe, biomedical researchers have rapidly launched an enormous number of clinical research studies aimed at testing diagnostic technologies, therapies, and vaccines. By April 2021, more than 3000 clinical trials related to COVID-19 had been registered with ClinicalTrials.gov [1]. The rapid, complete, and unbiased public dissemination of results from these trials is critical to enabling medical providers and public health leaders to make decisions that minimize new infections and optimize outcomes for infected patients [2]. While little is known about the dissemination of results from clinical trials performed during pandemic events, there is substantial evidence that trial results in general are often not made public in a timely fashion, prompting the World Health Organization (WHO), International Committee of Medical Journal Editors (ICMJE), and numerous other stakeholders to emphasize the importance of rapidly making trial data publicly available [3–6]. Specifically, the WHO has established standards for disseminating findings from clinical trials which require both the posting of results to a trial registry within 12 months of study completion and publication of trial results in a peer reviewed journal within 24 months of study completion [3].

Because of both the large scale and sporadic nature of pandemics, the process for performing clinical trials during these events differs in important ways from the process for most other diseases. Public recognition of the urgent need for biomedical research, motivation of regulators to facilitate trial activities, and interest in publishing among medical journals may all be heightened during pandemics [7, 8]. As a result, the timeline for biomedical research may be compressed, meaning that clinical trials are performed in human subjects based on limited pre-clinical data and without first performing observational or pilot studies. Additionally, when the pandemic is from a new disease, decisions about these trials are often made with a limited understanding of the natural history and mechanisms underlying the illness. Public opinion and political leaders may also create pressure on funders and investigators to focus on specific treatments, sometimes in the absence of compelling pre-trial evidence [9]. The collective impact of these various influences on the time frame for which results from studies are made available is poorly understood. Importantly, these factors also highlight the critical public health importance of publicly and promptly releasing trial data during pandemic events.

In order to better understand the dissemination of knowledge from clinical trials performed during pandemic events, we assessed the publication of outcome data from trials performed during three recent global-scale infectious disease events: H1N1 influenza in 2009, Ebola in 2014, and Zika in 2016. Each of these pandemics was labeled a Public Health Emergency of International Concern (PHEIC) by the World Health Organization, and each was caused by viruses for which disease-specific treatments were either unavailable or of limited proven efficacy, mirroring conditions during the current coronavirus outbreak [10]. In each of these cases the international biomedical research community also responded by launching a large number of clinical trials [11].

We examined the availability of outcome data from clinical trials launched during the H1N1, Ebola, and Zika outbreaks in order to test the hypothesis that results dissemination did not occur rapidly enough to meet established WHO standards for a substantial proportion of human-subject trials completed during these three pandemic events.

## Methods

We performed a cross-sectional analysis of clinical trials launched in response to the H1N1, Ebola, and Zika PHEIC events to assess outcome reporting patterns among trials performed during these pandemics.

### Trial eligibility

We defined a clinical trial according to the definition used by the WHO: any research study that prospectively assigns human participants or groups of humans to one or more health-related interventions to evaluate the effects on health outcomes [12]. Clinical trials were eligible for inclusion if they addressed the prevention, diagnosis, or treatment of organisms responsible for the 2009 H1N1 influenza outbreak, the 2014 Ebola outbreak, or the 2016 Zika outbreak. In order to be eligible, trials must have initiated enrollment no earlier than one year prior to the beginning of the PHEIC declaration, and no later than one year after conclusion of the PHEIC, as defined by the World Health Organization. Because clinical trial registries were used to identify eligible trials, registration in ClinicalTrials.gov or one of the Primary Registries in the WHO Registry Network was also required [13]. Trials with a registered completion date which was after February 2019 were excluded to allow time for data analysis and dissemination of results among the included sample. Trials were also excluded if the registry entry indicated that the trial was halted prior to enrolling any participants.

### Trial identification

We identified eligible trials by searching ClinicalTrials.gov and the WHO International Clinical Trials Registry Platform (ICTRP) for all trials, as well as the Pan African Clinical Trial Registry for Ebola, and the Brazilian Clinical Trials Registry, Cuban Public Registry of Clinical Trials and Peruvian Clinical Trials Registry for Zika. An investigator experienced in the use of trial registries (CWJ) searched each registry using key words and MeSH terms relevant to each PHEIC event (Supplementary Appendix A). After these searches, we removed duplicate trial entries and reviewed the full text of the remaining registry records to assess eligibility. For each included trial, we then downloaded a dataset from the relevant registry database containing key methodologic and logistical information, including the intervention being tested, funding source, and enrollment status.

### Search for trial results and publications

For each included trial we determined whether results had been posted directly to the registry, and the date on which the results were posted. We also performed a comprehensive literature search to identify peer reviewed publications describing trial results. Some registries, including ClinicalTrials.gov, encourage investigators to update registry entries with a link to PubMed-indexed manuscripts containing trial results. Additionally, ClinicalTrials.gov uses each entry’s unique trial identification number (NCT number) to automatically search for and link to relevant PubMed entries. We reviewed each linked publication to determine whether it contained results from the relevant trial. For registry entries without a linked publication containing trial results, we conducted literature searches to identify relevant publications. The publication search strategy was created in consultation with a team of health-sciences research librarians, and involved searching MEDLINE via PubMed, Google Scholar, and EMBASE by trial registration number, keywords, trial title, and investigator name for manuscripts matching each included trial. Study investigators performed three independent searches before a trial was considered unpublished, including searches by the study’s Principal Investigator (CWJ) and a health-sciences research librarian for each trial. The final assessment of results within the registry and the final literature search occurred in September and October 2020.

### Matching of registered trials and publications

We determined whether registry entries and publications identified by our search strategy matched by comparing the study title, trial design, interventions, number of participants, recruitment dates, study locations, investigators, and funding sources. A trial was considered published if we identified a peer-reviewed manuscript reporting un-pooled outcome data from the trial in question. Therefore, manuscripts that only described study methods without reporting trial results and those reporting results only as part of a pooled analysis were not considered to contain published results.

### Study outcomes

We utilized the standards for disseminating clinical trial results established by the WHO as the basis for the outcomes of the present study. These standards require both the posting of results to a trial registry within 12 months of study completion and publication of trial results in a peer reviewed journal within 24 months of study completion [3]. The primary outcome for this study was the presence or absence of publicly available results meeting either one of these established standards. To maintain consistency with the WHO standards, we did not consider non-peer reviewed publications or conference abstracts to fulfill the publication requirement. Additionally, we considered each trial’s completion date to be the trial’s primary completion date, or the final date on which data were collected for the trial’s primary outcome measure.

Our secondary outcomes include full compliance with the WHO results dissemination standards, defined by meeting both the 12-month deadline to post results in a trial registry and the 24-month publication standard. We also report publication status in a peer reviewed journal at any time (regardless of whether publication met the WHO’s 24-month standard), and availability of results in a trial registry at any time (regardless of whether this met the 12-month standard). Finally, we report the elapsed time between trial completion and public availability of results in a peer reviewed publication and in a trial registry.

### Subgroup analyses

We grouped eligible registered trials into prespecified subgroups according to the disease being studied (H1N1 influenza, Ebola, Zika), intervention type (drug/biologic, vaccine, other), funding source (industry, federal government, university, other), and trial phase. For drug trials containing at least one intervention arm and one control arm, we reviewed published manuscripts and registered outcome data to classify the study results as positive, negative, or neutral. Superiority trials were considered positive if they reported a statistically significant primary outcome result favoring the intervention arm, based on the significance threshold chosen by the study investigators, negative if they found a significant primary outcome result favoring the control group, and neutral if there was no difference in the primary outcome. Finally, we performed a sensitivity analysis involving only those trials with a registered enrollment status indicating that enrollment had concluded and would not re-open.

### Data analysis

Results describing categorical data are presented using descriptive statistics. We describe continuous data using medians and interquartile ranges (IQR). We used Kaplan–Meier methods to estimate the cumulative percentage of trials having publicly available outcome data over time, censoring unpublished trials on the date of the final unsuccessful manuscript search. We also report study outcomes according to our prespecified subgroups using descriptive statistics. We report the number of cases with missing data when relevant. These analyses were performed using SPSS version 27 (IBM Corp, Armonk NY).

## Results

**Fig. 1:**
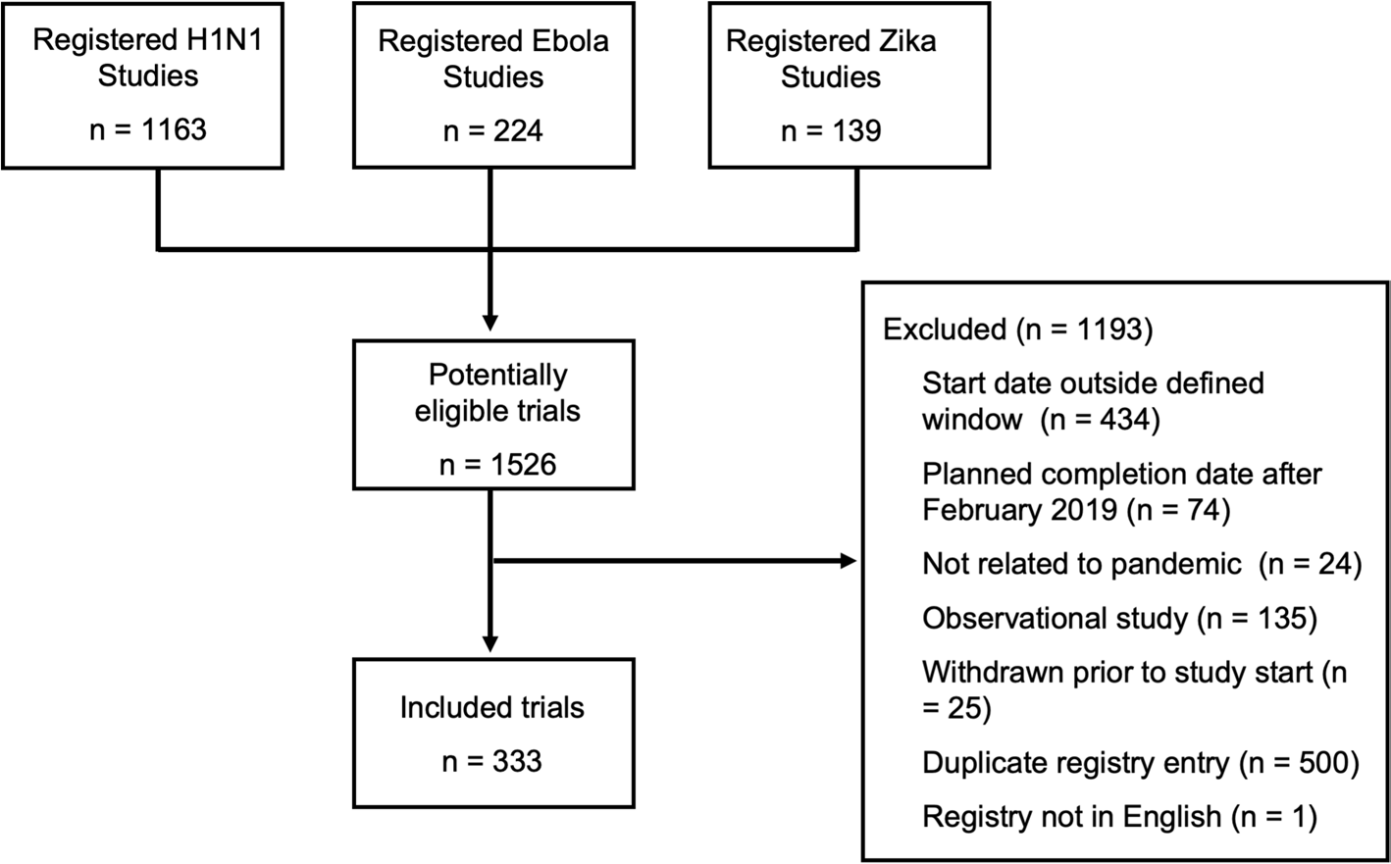
Flowchart of included trials

The initial registry queries generated 1526 potentially relevant registry records, of which 333 met eligibility criteria and were included in the analysis. These include 261 trials related to the 2009 H1N1 influenza outbreak, 60 trials related to Ebola in 2014, and 12 trials related to Zika in 2016 (Fig. 1, Appendix B). The majority of trials (*n *= 206, 62%) were industry funded (Table 1). Registry records indicated that enrollment had been completed normally in 286 (86%) of trials and had halted early or been suspended in 20 (6%) trials. We could not verify that enrollment had stopped for the remaining 27 (8%) trials, though in each case the anticipated primary completion date in the registry was before March 2019.

**Table 1:** Characteristics of included clinical trials

Trial Characteristics; *n* (%) unless noted	Total (*n *= 333)	H1N1 (*n *= 261; 78%)	Ebola (*n *= 60; 18%)	Zika (*n *= 12; 4%)
Number of participants; median (IQR)	175 (89–445)	200 (117–468)	97 (53–322)	49 (37–143)
Funding^a^				
Industry	206 (62%)	170 (65%)	31 (52%)	5 (42%)
Federal Government	94 (28%)	56 (22%)	34 (57%)	4 (33%)
University	89 (27%)	62 (24%)	23 (38%)	4 (33%)
Other	35 (11%)	24 (9%)	11 (18%)	0 (0%)
Trial Phase				
Phase I	59 (18%)	20 (8%)	30 (50%)	9 (75%)
Phase II^b^	96 (29%)	82 (31%)	14 (23%)	0 (0%)
Phase III^c^	79 (24%)	74 (28%)	5 (8%)	0 (0%)
Phase IV	54 (16%)	54 (21%)	0 (0%)	0 (0%)
Other	45 (14%)	31 (12%)	11 (18%)	3 (25%)
Intervention Type				
Drug/biologic	44 (13%)	30 (11%)	14 (23%)	0 (0%)
Vaccine	276 (83%)	225 (86%)	42 (70%)	9 (75%)
Other	13 (4%)	6 (2%)	4 (7%)	3 (25%)
Source Register				
ClinicalTrials.gov	263 (79%)	204 (78%)	48 (80%)	11 (92%)
European Union Clinical Trials Register	32 (10%)	32 (12%)	0 (0%)	0 (0%)
Clinical Trials Registry of India	11 (3%)	10 (4%)	0 (0%)	1 (8%)
Pan African Clinical Trials Registry	6 (2%)	0 (0%)	6 (10%)	0 (0%)
Japan Primary Registries Network	6 (2%)	4 (2%)	2 (3%)	0 (0%)
Other Registry	15 (5%)	11 (4%)	4 (7%)	0 (0%)
Recruiting Status in Registry				
Enrollment completed	286 (86%)	225 (86%)	51 (85%)	10 (83%)
Enrollment not complete	11 (3%)	8 (3%)	3 (5%)	0 (0%)
Stopped early	16 (5%)	14 (5%)	2 (3%)	0 (0%)
Suspended/not currently enrolling	4 (1%)	2 (1%)	1 (2%)	1 (8%)
Unknown status	16 (5%)	12 (5%)	3 (5%)	1 (8%)

^a^ Categories are not mutually exclusive

^b^ Includes trials labeled “phase I/II”

^c^ Includes trials labeled “phase II/III”

Of the 333 included trials, 139 (42%) either had results available in the trial registry within 12 months of the primary study completion date or had results available in a peer-reviewed publication within 24 months of completion, thereby meeting either of the standards for results dissemination established by the WHO. Five of the included trials (2%) met both of these standards and were therefore in full compliance with the WHO requirements (Table 2). Fifty-nine trials (18%) had no results available in either a registry or peer reviewed publication at the time of our search in the fall of 2020.

**Table 2:** Availability of outcome data for included trials

Outcome; n (%) unless noted	Total (*n *= 333)	H1N1 (*n *= 261; 78%)	Ebola (*n *= 60; 18%)	Zika (*n *= 12; 4%)
**Registry Outcomes**				
Results available in registry within 12 months of primary completion date	15 (5%)	11 (4%)	4 (7%)	0 (0%)
Results available in registry at any time	158 (47%)	144 (55%)	13 (22%)	1 (8%)
No results in registry	175 (53%)	117 (45%)	47 (78%)	11 (92%)
Time from completion to results in registry among trials with registry results, months; median (IQR)	41.5 (16–76)	34 (16–76)	29 (14–43)	n/a
**Publication Outcomes**				
Published within 24 month of primary completion date	129 (39%)	94 (36%)	29 (48%)	6 (50%)
Published at any time	228 (69%)	181 (69%)	41 (68%)	6 (50%)
Not published	105 (32%)	80 (31%)	19 (32%)	6 (50%)
Time from completion to publication among published trials, months; median (IQR)	21 (9–34)	23 (13–34)	16 (0–34)	n/a
**Combined Registry and Publication Outcomes**				
Registry has results within 12 months of completion or published within 24 months	139 (42%)	100 (38%)	33 (55%)	6 (50%)
Registry has results within 12 months of completion and published within 24 months	5 (2%)	5 (2%)	0 (0%)	0 (0%)
Published and results in registry	112 (34%)	100 (38%)	11 (18%)	1 (8%)
Published but no results in registry	116 (35%)	81 (31%)	30 (50%)	5 (42%)
Not published but results are in registry	46 (14%)	44 (17%)	2 (3%)	0 (0%)
Not published and no results in registry	59 (18%)	36 (14%)	17 (28%)	6 (50%)

**Fig. 2:**
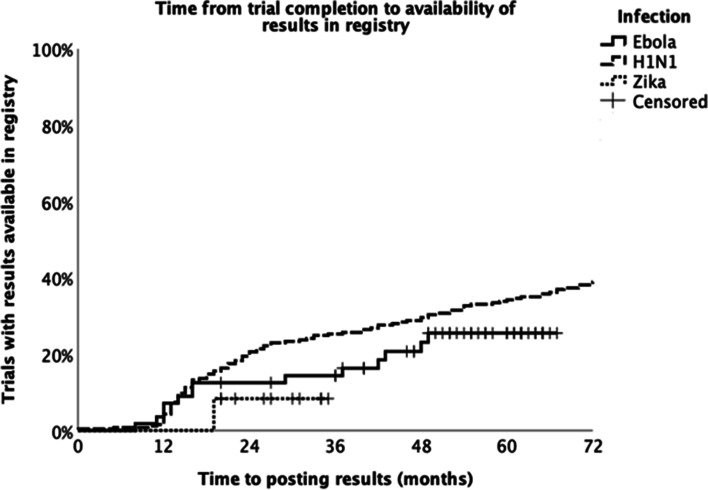
Kaplan–Meier analysis showing time elapsed between trial completion and posting of results to a trial registry

Results were uploaded to a registry within 12 months of study completion, 24 months, and at any time point respectively for 15 (5%), 61 (18%), and 158 (47%) trials. Among trials with available results, the median delay between the primary study completion date and the availability of results on a registry was 42 months (IQR 16–76 months); this delay was at least 5 years for 57 trials (37% of those with results) (Fig. 2).

**Fig. 3:**
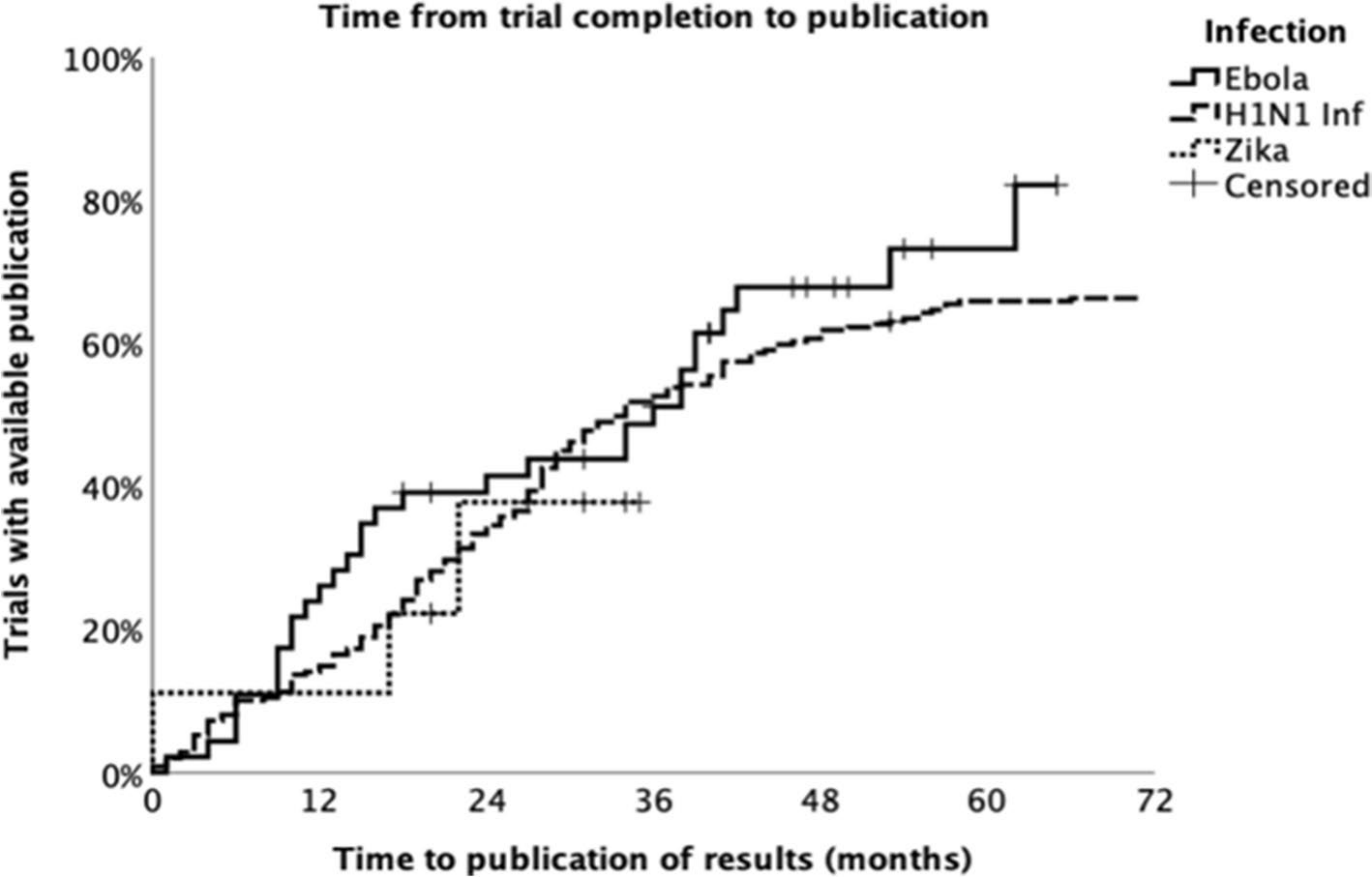
Kaplan–Meier analysis showing time elapsed between trial completion and publication of a peer-reviewed manuscript containing outcome data

Peer-reviewed manuscripts were published within 12 months, 24 months, and at any time point following trial completion for 71 (21%), 129 (39%), and 228 (68%) trials. For trials which were published at any time, the median elapsed time between trial completion and publication was 21 months (IQR 9–34 months) (Fig. 3). In total, results were available within 24 months of study completion in either the trial registry or a peer reviewed publication for 166 trials (50%).

A lower proportion of Zika trials (8%) and Ebola trials (22%) had results uploaded to a registry at any time than H1N1 trials (55%). The overall proportion of published trials was similar across the different diseases, though time to publication was substantially faster for both Zika and Ebola trials than for H1N1 trials (Table 3). The median delay between trial completion and availability of results in the trial registry was shorter for federally funded trials (14 [IQR 13–24] months) than for non-federally funded trials (52 [IQR 22–80] months). Phase 3 trials were published after a median delay of 28 (IQR 18–34) months following trial completion, which was slower than non-phase 3 trials (18 [IQR 6–33.5] months). Of the 44 trials assessing non-vaccine drugs or biologics, 16 (36%) had no results available, seven (16%) reported positive results favoring the intervention group, 14 (32%) found no evidence for either benefit or harm of the intervention, none reported a negative result, and seven (16%) did not include a control condition. Five of the seven positive trials (71%) were either published within 24 months or had registered results within 12 months, compared with five of 14 neutral trials (36%).

**Table 3:** Availability of outcome data by trial subgroup

Trial characteristic	Total trials; n	Registry has results within 12 months of trial completion; n (%)	Months elapsed from completion to results in registry; median (IQR)	Publication within 24 months of trial completion; n (%)	Months elapsed from completion to publication; median (IQR)
Total	333	15 (5%)	42 (16–76)	129 (39%)	21 (9–34)
Infection					
H1N1	261	11 (4%)	45 (18–78)	94 (36%)	23 (12–34)
Ebola	60	4 (7%)	16 (12–43)	29 (48%)	11 (-2–34)
Zika	12	0 (0%)		6 (50%)	-3 (-11–18)
Funding Source					
Industry	206	8 (4%)	48 (22–80)	71 (35%)	24 (10–34)
Federal Government	94	7 (7%)	14 (13–24)	47 (50%)	17 (6–28)
University	89	2 (2%)	51 (18–75)	49 (55%)	15 (6–27)
Other	35	2 (6%)	16 (6–32)	16 (46%)	10 (1–28)
Trial Phase					
Phase I	59	1 (2%)	42 (16–49)	24 (41%)	17 (-4–40)
Phase II	96	11 (12%)	25 (13–73)	43 (45%)	19 (5–28)
Phase III	79	1 (1%)	43 (21–80)	23 (29%)	28 (18–34)
Phase IV	54	1 (2%)	65 (24–85)	21 (39%)	19 (12–36)
Other	45	1 (2%)	21 (14–79)	18 (40%)	18 (9–38)
Intervention Type					
Drug/biologic	44	0 (0%)	32 (19–59)	17 (39%)	19 (9–34)
Vaccine	276	15 (5%)	43 (16–78)	108 (39%)	22 (9–34)
Other	13	0 (0%)		4 (31%)	22 (13–48)
Recruiting Status in Registry					
Enrollment completed	286	15 (5%)	41 (16–76)	118 (41%)	21 (9–34)
Enrollment not complete	11	0 (0%)		2 (18%)	41 (16–73)
Stopped early	16	0 (0%)	49 (21–75)	2 (13%)	21 (7–41)
Suspended/not currently enrolling	4	0 (0%)		1 (25%)	
Unknown status	16	0 (0%)		6 (38%)	13 (5–25)

A total of 302 trials (91%) had an enrollment status in the registry that indicated enrollment had either been completed as scheduled or terminated early with no plans to begin again. The availability of outcome data was similar for these trials as compared to the group of all included trials, with 130 (43%) either having registered results available within 12 months or a peer-reviewed publication within 24 months of trial completion. Five completed trials (2%) met both of these criteria.

## Discussion

We performed a cross-sectional analysis of trials registered in publicly accessible trial registries which assessed interventions studied in response to the 2009 H1N1, 2014 Ebola, and 2016 Zika pandemics. Our findings demonstrate that nearly one in five trials had no available outcome data in either a publication or trial registry. Furthermore, among trials with available results, most failed to meet either one of the two standards established by the WHO for disseminating trial results in a timely fashion. Only 2% of trials met both of the WHO standards.

The WHO has defined maximum delays of 12 months between trial completion and posting of results to the trial registry and 24 months between completion and publication in a peer reviewed journal as standards which nearly all clinical trials should meet [3]. Because registered results and peer reviewed manuscripts often contain complementary data, trial sponsors are expected to meet both of these standards [14, 15]. Additionally, for most clinical trials within the United States, a trial’s sponsor is required to submit results to the registry within 12 months after the date of final data collection for the primary outcome has occurred, though extensions can be obtained for trials involving unapproved investigational products or for trials seeking a new use of an approved product [16]. Sponsors that fail to meet this requirement can be subject to fines of up to $10,000 per day that they remain out of compliance [17]. Despite the public declaration of these requirements, we find that results from pandemic trials often remain unavailable even after lengthy delays. Many factors impact the feasibility of performing clinical research during pandemics, including fluctuating case numbers, funding availability, and available research infrastructure. These challenges to performing clinical trials during pandemics emphasize the importance of effectively sharing results from those trials which are able to be conducted. Failure to publicly disseminate clinical trial data is likely to be particularly harmful to patient outcomes during pandemic events involving novel or previously obscure pathogens. Often very little disease-specific clinical data is available to guide care during the early phases of these events, and therefore delays in access to trial results have the potential to delay the adoption of evidence-based treatment regimens.

Prior work has shown that clinical trials are often conducted without subsequent publication of trial results. Studies specifically assessing the publication of vaccine trials and trials involving rare diseases have shown that in both cases approximately seven in 10 are unpublished two years after completion, and that approximately three in 10 trials still remain unpublished four years after completion [18, 19]. Our findings show that pandemic trials are slightly more likely to be published within two years, but that long-term rates of nonpublication are similar. These high rates of delayed publication and nonpublication are problematic for any group of trials, but are particularly concerning for pandemic trials. Timely release of knowledge gained from research conducted during pandemics is essential to minimize the spread and impact of the disease. Making results available from these studies is also important because due to the availability of patients and research funding, pandemics present unique opportunities to perform large-scale clinical studies of diseases that are present for a limited period of time, but nonetheless have the potential to recur.

There are several possible solutions to the problem described. One solution that has played out in a public manner during the coronavirus pandemic is the utilization of press releases and preprints as methods of rapidly disseminating study findings [20]. Owing to the magnitude of the public health crisis and possibly influenced by the financial implications of reporting a successful treatment or vaccine, a number of investigators and companies have made results available in this manner even prior to trial completion or full internal review of results [21–24]. This approach goes a long way to accelerate the process by which findings are shared with the public. However, these releases occur without peer review and without full release of study data, and the degree to which press releases and preprints are inaccurate or offer a biased interpretation of results is not known. Additionally, these mechanisms have the potential to be particularly misleading if they are utilized more frequently for trials with positive results, thereby producing a type of publication bias in which neutral or negative trials are subject to delays in dissemination compared to positive trials. Future work is needed to characterize omissions, errors, and bias in press releases and preprints and, if indicated, policies are needed to ensure results are comprehensive, accurate, and unbiased. Other solutions to the problem of delays in the release of clinical trial results could include enforcement of financial penalties for delaying release of results, review of compliance with these requirements during regulatory review of new drugs or devices, review of compliance during grant review for future funding by investigators and sponsors, or systems that ensure the automatic release of data at a prespecified time point.

Several strengths and limitations of this work should be considered when interpreting the study results. We utilized a comprehensive publication search strategy designed by research librarians and physicians with expertise in working with both trial registries and publication databases, and trials were only classified as unpublished if no link to a publication was available on the registry website and no matching manuscript was identified after three search attempts. Despite this, it is possible that we failed to identify some relevant publications, particularly if these publications failed to report the trial’s unique registry identification number. Additionally, since 2005 the International Committee of Medical Journal Editors and WHO have required prospective trial registration, and many nations have also implemented registration requirements [25, 26]. Despite these requirements, some trials remain unregistered. It is unlikely, however, that trials which fail to comply with existing registration requirements are published more rapidly than registered trials. Furthermore, unlike Ebola and Zika, influenza has been the focus of numerous clinical trials independent of the 2009 H1N1 pandemic. Because our interest here is in the time frame for the release of results from research related to pandemics, we only included influenza trials with registry entries that specifically referenced H1N1 or pandemic influenza. Our analysis of trial results may be impacted by results availability bias, as we were only able to determine the direction of results (i.e., positive, neutral, negative) for those trials in which results had been publicly released. For most trials without public results, we do not know why the results were not made available. In some cases, this may have been because the investigators or funders did not want to publish a negative trial. In other cases, it may have been that the company funding the study had a change in priorities and decided to move on to other work [27, 28]. Our opinion, which is supported by regulations, is that if data are collected from participants then the results should be made publicly available. Additionally, few Zika trials were identified relative to the number of H1N1 and Ebola trials, which limits the generalizability of our findings. Finally, we did not analyze trials related to the 2014 polio PHEIC. Polio is different from the diseases examined here because an effective polio vaccine existed at the time of the PHEIC declaration, and the major focus around polio is focused on the policies and procedures to ensure widespread vaccination rather than clinical trials to generate new medical knowledge about prevention and treatment.

## Conclusions

We found that for clinical trials conducted during recent pandemics prior to COVID-19, delays in the release of data and publication of results are common, with half of trials having neither results posted to a registry after one year nor published in a peer reviewed journal two years after trial completion. The problem of delays in the availability of trial results was observed for all three pandemics studied and was also similar for both treatments and vaccine trials. Policies to ensure that comprehensive, accurate, and unbiased results of pandemic-related research are made available in a timely manner are needed.

## Supplementary Information

**Additional file 1: Appendix A.**

**Additional file 2 Appendix B.**

## Abbreviations

WHO: World Health Organization
COVID-19: Coronavirus 2019
ICMJE: International Committee of Medical Journal Editors
PHEIC: Public Health Emergency of International Concern
ICTRP: International Clinical Trials Registry Platform
IQR: Interquartile range
