# Supplementary material for: Delays in reporting and publishing trial results during pandemics: cross sectional analysis of 2009 H1N1, 2014 Ebola, and 2016 Zika clinical trials
Source: BMC Med Res Methodol. 2021 Jun 8;21:120. doi: 10.1186/s12874-021-01324-8 (PMC8185489; doi:10.1186/s12874-021-01324-8)
Supplement: Supplementary file 1 — Additional file 1: Appendix A. [file 12874_2021_1324_MOESM1_ESM.docx]

Search Terms:

H1N1 influenza search terms:

ClinicalTrials.gov: H1N1 OR "pandemic influenza" OR "pandemic flu"

ICTRP: H1N1 OR (pandemic AND influenza) OR (pandemic AND flu)

Zika search terms:

Zika

Ebola search terms:

Ebola OR Ebola virus
